# A comparative study protocol of external fixation versus volar plate in treating distal radius fracture

**DOI:** 10.1097/MD.0000000000023231

**Published:** 2020-12-11

**Authors:** Fuqiang Zhang, Yang Yang, Hui Zhang, Xiangli Luo

**Affiliations:** Department of Orthopaedics, Gansu Provincial Hospital, Lanzhou, Gansu, 730000, China.

**Keywords:** cohort study, external fixation, intra-articular distal radius fractures, open reduction and internal fixation, protocol

## Abstract

**Background:**

The superiority of the open reduction and internal fixation (ORIF) and external fixation remains uncertain owing to the limited sample size involved in the literature. This retrospective cohort research was implemented for the comparison of the efficiency of ORIF and external fixation utilizing the locked volar plating in treating the intra-articular fractures of distal radius. We hypothesized that compared with the external fixation, ORIF can improve the functional scores and reduce complications.

**Methods:**

We conducted a retrospective and single-center cohort trial that was approved by institutional review committee of Gansu Provincial Hospital. From June 2016 to July 2018, in our experiment, 178 patients with intra-articular fractures of the distal radius were recruited. Inclusion criteria in this cohort study were designed as follows: the age of patients is between 18 and 65 years, the patients with American Society of Anesthesiologists level I–III, and with the AO-type C3 or C2 fractures of distal radius confirmed by computed tomography scans, the patients with radiographic clinical follow-up for 1 year or >1 year. The patients participating in the trial would be divided into 2 groups: the patients treated via the external fixation and the patients treated by the ORIF utilizing volar plate. The main functional results were the grip strength and the range of motion of wrist. Radiographic measurement and complications were also evaluated in our study.

**Results:**

This study protocol will guide and clarify our assignments, and the final outcomes and conclusion will further enrich the clinical knowledge in the literature.

**Trial registration:**

This study protocol was registered in Research Registry (researchregistry6116).

## 1 Introduction

Distal radius fractures account for 44% of all kinds of the forearm and hand fractures, which is the most familiar kind of upper limb fractures and lead to a serious problem of public health.^[1–4]^ In China alone, about 580 thousand of distal radius fractures occurred in 2014, and the incidence rate of distal radius fractures is on the rise both domestically and internationally.^[5,6]^

For decades, many different treatments such as external fixation, cast immobilization, and open reduction and internal fixation (ORIF) with the dorsal or volar plates have been tried to treat the intra-articular fractures of distal radius.^[7–10]^ To date, there is no consensus on the best treatment of intra-articular fractures of the distal radius. The technology of external fixation has been developed, with multiple studies have shown that the anatomical results and functional score of the external fixator were better than those of the closed reduction and casting.^[11–13]^ The advantages of ORIF contain the stable fragments subchondral fixation in the joint and early postoperative active wrist movement.^[14]^ Many investigations have concluded that the better normal joint anatomy restoration is essential to obtain optimal functional outcomes and prevent posttraumatic arthritis (especially comminuted intra-articular fractures).^[15,16]^ Several previous cohort studies have been conducted for the comparison of the results between ORIF and external fixation and closed reduction. Due to the variability and complexity of a single series of fractures or the distinct techniques utilized between the cases, the clear conclusions are difficult to be obtained.^[17–19]^

The superiority of the ORIF and external fixation remains uncertain owing to the limited sample size involved in the literature. This retrospective cohort research was implemented for the comparison of the efficiency of ORIF and external fixation utilizing the locked volar plating in treating the intra-articular fractures of distal radius. We hypothesized that compared with the external fixation, ORIF can improve the functional scores and reduce complications.

## 2 Materials and methods

### 2.1 Patients

We conducted a retrospective and single-center cohort trial that was approved by institutional review committee of Gansu Provincial Hospital (L2020-08-18). From June 2016 to July 2018, in our experiment, 178 patients with intra-articular fractures of the distal radius were recruited. The patients participating in the trial would be divided into 2 groups: the patients treated via the external fixation and the patients treated by the ORIF utilizing volar plate. The trial protocol was registered with the Research Registry, and the registration number researchregistry6116. Inclusion criteria in this cohort study were designed as follows: the age of patients is between 18 and 65 years, the patients with American Society of Anesthesiologists level I–III, and with the AO-type C3 or C2 fractures of distal radius confirmed by computed tomography scans, the patients with radiographic clinical follow-up for 1 year or >1 year. Patients suffering from any other related injuries/fractures, bilateral distal bone fractures, open fractures, and concomitant infectious diseases were not included in the study. Patients with stable fractures, younger than 25 or older than 65 years old, or concomitant any systemic disease were also excluded from the study.

### 2.2 Intervention and control techniques

In this present experiment, the operations were carried out by the experienced surgeons or the researchers under their direct supervision.

#### 2.2.1 External fixation group

For fractures with minor impacted fragments, the fluoroscopically guided continuous slight traction was implemented to achieve and keep reduction. Afterwards, the external fixator was fixed directly to the second or third metacarpal bone and the radius, respectively utilized the 3-mm pins and 4-mm Schanz pins. For the large fractures of articular surface or unsatisfactory reduction of fractures where there is significant displacement and collapse, a small incision needed to be made at the volar of radius, and the collapsed fragments were raised with the periosteum elevator. If the serious impacted fragments or the bone detect was found, allograft bone or allograft was implanted.

#### 2.2.2 ORIF group

In ORIF group, the reduction was improved finally by operating the bone fragment directly with an anterior approach, and locking volar plate needed to be fixed before the fixator was removed. Despite all the metaphyseal screws are locked on distal bone, one screw or more screws are locked on proximal bone. The volar splint is maintained on the wrist for 6 weeks after surgery, after that the re-education was required.

### 2.3 Outcome measures

The main functional results were the grip strength and the range of motion (ROM) of wrist. The ROM containing internal rotation, extension supination, and the flexion of wrist was measured by goniometer. A grip strength could be measured with the Jamar dynamometer. The comparison between all of these determinations and contralateral uninjured wrist was conducted. Standard posterior anterior and lateral radiographs were used to assess the radiographic measurement containing the radial length, radial inclination, volar tilt, and others.

Complications including implant failure, digital stiffness, tendon contractures, infection, nerve pathology, and tendinitis were assessed and then recorded. The analysis of complications related to bone included the early postoperative arthritis, nonunion, and delayed union. At the aim of extracting these data for the study purpose, the fractures could be classified as the delayed unions if there is no radiographic evidence of trabecular bridging at the fracture site at 4 months. Fractures were categorized as nonunion if they continued to show no healing at 6 months. If the patient complained of the arthritis pain and reveals obvious stenosis of the joint space, as well as osteophyte formation at the wrist or radio-ulnar joint, it would be considered to have symptomatic postoperative arthritis (Table 1).

**Table 1:** Postoperative outcomes.

Outcomes	Group A	Group B	*P* value
ROM			
Grip strength			
Radiographic outcomes			
Length of hospital stay			
Complications			

ROM = range of motion.

### 2.4 Statistical analysis

The chi-squared test was utilized for the evaluation of the differences in the categorical variables containing comorbidities, sex, and complications. The Student *t* test was applied for the assessment of differences in the continuous variables, involving range of motion, age, grip strength, and radiological measurements. For all the statistical tests, the significance level was set as *P* = .05. And all the analyses were carried out by the SAS software (version 9.3 and SAS Enterprise Guide 6.1; SAS Institute; New York, United States ).

## 3 Discussion

The intra-articular distal radius fractures is a kind of unstable, complex, and high-energy injuries, and the optimal treatment is still controversial. showed that if the intra-articular steps and gap deformities were minimized, external fixation could provide faster functional recovery and better functional results within 2 years compared with ORIF. However, the other studies showed no difference between external fixation and ORIF. This retrospective cohort research was implemented for the comparison of the efficiency of ORIF and external fixation utilizing the locked volar plating in treating the intra-articular fractures of distal radius. We hypothesized that compared with the external fixation, ORIF can improve the functional scores and reduce complications.

## Author contributions

**Conceptualization:** Xiangli Luo.

**Data curation:** Fuqiang Zhang, Yang Yang.

**Formal analysis:** Fuqiang Zhang, Yang Yang.

**Funding acquisition:** Xiangli Luo.

**Investigation:** Fuqiang Zhang, Yang Yang.

**Methodology:** Hui Zhang.

**Project administration:** Xiangli Luo.

**Resources:** Xiangli Luo.

**Software:** Fuqiang Zhang, Hui Zhang, Yang Yang.

**Supervision:** Xiangli Luo.

**Validation:** Fuqiang Zhang, Hui Zhang, Yang Yang.

**Visualization:** Yang Yang, Hui Zhang.

**Writing – original draft:** Fuqiang Zhang, Yang Yang.

**Writing – review & editing:** Xiangli Luo.
